# Representation and decoding of bilateral arm motor imagery using unilateral cerebral LFP signals

**DOI:** 10.3389/fnhum.2023.1168017

**Published:** 2023-06-14

**Authors:** Jiafan Lin, Dongrong Lai, Zijun Wan, Linqing Feng, Junming Zhu, Jianmin Zhang, Yueming Wang, Kedi Xu

**Affiliations:** ^1^Qiushi Academy for Advanced Studies, Zhejiang University, Hangzhou, China; ^2^Zhejiang Provincial Key Laboratory of Cardio-Cerebral Vascular Detection Technology and Medicinal Effectiveness Appraisal, Key Laboratory for Biomedical Engineering of Ministry of Education, School of Biomedical Engineering and Instrument Science, Zhejiang University, Hangzhou, China; ^3^Zhejiang Lab, Hangzhou, China; ^4^Department of Neurosurgery, Second Affiliated Hospital, School of Medicine, Zhejiang University, Hangzhou, China; ^5^State Key Lab of Brain-Machine Intelligence, Zhejiang University, Hangzhou, China; ^6^College of Computer Science and Technology, Zhejiang University, Hangzhou, China; ^7^MOE Frontier Science Center for Brain Science and Brain-Machine Integration, Zhejiang University, Hangzhou, China

**Keywords:** bilateral decoding, brain computer interface, local field potential, motor imagery, unilateral motor cortex

## Abstract

**Introduction:**

In the field of upper limb brain computer interfaces (BCIs), the research focusing on bilateral decoding mostly based on the neural signals from two cerebral hemispheres. In addition, most studies used spikes for decoding. Here we examined the representation and decoding of different laterality and regions arm motor imagery in unilateral motor cortex based on local field potentials (LFPs).

**Methods:**

The LFP signals were recorded from a 96-channel Utah microelectrode array implanted in the left primary motor cortex of a paralyzed participant. There were 7 kinds of tasks: rest, left, right and bilateral elbow and wrist flexion. We performed time-frequency analysis on the LFP signals and analyzed the representation and decoding of different tasks using the power and energy of different frequency bands.

**Results:**

The frequency range of <8 Hz and >38 Hz showed power enhancement, whereas 8–38 Hz showed power suppression in spectrograms while performing motor imagery. There were significant differences in average energy between tasks. What’s more, the movement region and laterality were represented in two dimensions by demixed principal component analysis. The 135–300 Hz band signal had the highest decoding accuracy among all frequency bands and the contralateral and bilateral signals had more similar single-channel power activation patterns and larger signal correlation than contralateral and ipsilateral signals, bilateral and ipsilateral signals.

**Discussion:**

The results showed that unilateral LFP signals had different representations for bilateral motor imagery on the average energy of the full array and single-channel power levels, and different tasks could be decoded. These proved the feasibility of multilateral BCI based on the unilateral LFP signal to broaden the application of BCI technology.

**Clinical trial registration:**

https://www.chictr.org.cn/showproj.aspx?proj=130829, identifier ChiCTR2100050705.

## 1. Introduction

Brain computer interface (BCI) provides a direct communication connecting the brain and external devices (Kawala-Sterniuk et al., 2021). A number of studies have shown that the kinematic information of limbs can be extracted from the neural signals of the motor cortex (Vilela and Hochberg, 2020). Regardless of whether limbs are in motion or motor imagery, these neural signals show different representation patterns for different movements (Miller et al., 2010). Through the decoding methods, external devices can be controlled such as computer cursors and robotic limbs (Hatsopoulos and Donoghue, 2009; Vilela and Hochberg, 2020).

The research of neural signal during limb movement has often focused on the contralateral cortex. After a number of studies have proved that the movement types, movement trajectories and other parameters can be represented and decoded by signals recorded from the contralateral cortex, some scholars turned their attention to ipsilateral neural signals. Cisek et al. (2003) demonstrated that when monkeys move their arms in the center-out tasks, 55% of the neurons in primary motor cortex (M1) are tuned during the movement of either contralateral or ipsilateral arm. However, the preferred directions of these neurons were different between two arms and there was a greater discharge rate during contralateral arm movement. Ganguly et al. (2009) found that when monkeys use exoskeleton to do center-out tasks, it is feasible to use spike activity to continuously decode the kinematic information of the ipsilateral arm. Bundy et al. (2018); Bundy and Leuthardt (2019) found that the electrocorticography (ECoG) signals in ipsilateral human cortex encode the three-dimensional arm movement kinematics. Furthermore, a BCI was implemented based on ipsilateral neural signals. Willett et al. (2020) used the spike signals of paralyzed patients, which were recorded from the hand knob area in one cerebral hemisphere, to successfully decode different contralateral and ipsilateral attempted movements of different limbs, namely, realized the multi-movement classification of limbs on both sides using unilateral spike signals. Some studies have further explored the relationship between neural signals in the contralateral and ipsilateral cortex. Ames and Churchland (2019) reported that the standard deviation of the firing rate across times and conditions showed no difference when the same task was performed by one arm of monkeys versus the other. Similarly, the muscle activity of one arm could be decoded equally well from the signals on either side of cerebral hemispheres (Ames and Churchland, 2019). However, the information in the two cerebral hemispheres were very different at the level of single neurons. For certain neurons, the activations caused by the two arms were essentially irrelevant. Heming et al. (2019) found that the representations of two limbs in primary motor cortex are independent, neural activity for the contralateral limb can be extracted without interference from the ipsilateral limb.

There are also some studies that have explored the representation of neural signals when both arms are moving simultaneously. Donchin et al. (2001, 2002) found that compared with unilateral arm movement, the activation of neural signals is not the same in bilateral arm movement, whether it is movement-evoked potentials or single-unit activity. Furthermore, this phenomenon could not be explained by the linear combination of unilateral arm movements. Although the neural signals of unilateral and bilateral arm movements have been shown to be different, there are less studies focusing on differentiating neural signals resulted from ipsilateral, contralateral, and bilateral movements. Choi et al. (2018) implemented a four-category classification for monkeys, which distinguished the four tasks of rest state and movements of pressing a button using left, right and both hands. Ifft et al. (2013) designed a BCI that allowed monkeys to control the movement of two robotic arms simultaneously. However, these research of bilateral movements is all based on the neural signals from two cerebral hemispheres. It is not clear whether the signal from unilateral cerebral cortex can be used to distinguish the movements of ipsilateral, contralateral and bilateral arms. In addition, due to the high-precision and spatiotemporal resolution characteristics of spike signals, most studies focusing on ipsilateral and bilateral movements are based on spikes (Collinger et al., 2013; Wodlinger et al., 2014; Willett et al., 2020; Dongrong et al., 2023). Local field potential (LFP) signals have also been proven to contain much movement information and show performance comparable to but lower than spikes (Heldman et al., 2006; Nason et al., 2020). In addition, the stability of LFP is much better than that of spike (Jackson and Hall, 2017). Wang et al. (2014) demonstrated that LFP can still decode kinematic information even when no spike can be recorded. Therefore, further studies are needed for the representation and decoding of contralateral, ipsilateral and bilateral movements using LFP signals to improve the application of BCI technology in long-term use and rehabilitation.

In terms of cerebral function related to movement, left and right hemispheres are not exactly the same. The left hemisphere reveals a better across-arm generalization while the right hemisphere concentrates more on the contralateral movements. In clinical trials, limb apraxia is the best known example of such left hemisphere dominance for action (Haaland, 2006). Haaland demonstrated that the incidence of limb apraxia is greater in patients with left hemisphere lesion than right hemisphere lesion (Haaland, 2006). In the analysis of unilateral movement, Merrick et al. (2022) also demonstrated the left hemisphere dominance. They analyzed the neural representations of contralateral and ipsilateral movements in two hemispheres in human and found stronger bilateral encoding in the left hemisphere (Merrick et al., 2022). In the analysis of bilateral movement, Blinch et al. (2019) found left hemisphere dominance in the bimanual symmetric movements.

In this study, the LFP signals recorded from unilateral cerebral cortex were used to characterize and decode the arm motor imagery of different laterality and regions. The tasks included ipsilateral, contralateral and bilateral movements of elbows and wrists. A paralyzed patient participated and was asked to look at the virtual arms on the front screen in the experiment. During the motion tasks, the participant needed to do motor imagery following the movement of virtual arms since he could not move his arms autonomously. The LFP signal was recorded from the primary motor cortex in the left cerebral hemisphere of the participant. First, the average power of different frequency bands of LFP signals were analyzed. Then demixed principal component analysis (dPCA) was used to find the two dimensions that separately characterize the laterality and region information. Second, the average power of 5 frequency bands in the range of 0.3–300 Hz were used as features to classify different tasks, respectively, and good performances were obtained. Finally, single-channel LFP signals were analyzed. The activation patterns in different tasks were explored, which helped to optimize the channels for classification. The signal correlations between different motion tasks were also analyzed, which showed the similarity of LFP responses for ipsilateral, contralateral, and bilateral movements. This study demonstrates the abundant information contained in LFP signal, and there is a great potential for it to characterize and decode movements of different laterality and body parts.

## 2. Materials and methods

### 2.1. Participant

The participant recruited for the experiment was a male paralyzed patient who suffered a traumatic cervical spine injury at C4 level. The body parts below his neck were unable to move. The participant had normal cognitive function and could well understand and complete all tasks (Wang et al., 2022). All the procedures were followed from the guide and approved by Institutional Review Board for Human Studies of the Second Affiliated Hospital of Zhejiang University School of Medicine. The participant and his immediate family members verbally agreed to participate in the experiment after detailed explanation, and the informed consent was signed by his legal representative.

### 2.2. Microelectrode implantation and neural recording

Two 96-channel Utah microelectrode arrays (4 mm × 4 mm, Utah Array with 1.5 mm length, Blackrock Microsystems, Salt Lake City, UT, USA) were implanted in the participant’s left cerebral cortex, one was placed in the hand knob area of M1, and the other was implanted in the arm area of M1 about 2 mm away from the previous one. The specific placement was shown in previous work (Qi et al., 2022). The participant was asked to image hand and elbow movement during fMRI scanning to determine the area before implantation. In this study, only the signals recorded from the array in arm area were analyzed. The other array could only record a few spike signals, the neural signals might not be well recorded.

Neural signals were recorded with the Neuroport system (Blackrock Microsystems). The LFP signals were recorded at a sample rate of 1 kHz for each channel. The 50 Hz interference induced by the electrical power system in the signal was removed by software filtering during the recordings, thus the 50 Hz notch filter was no longer used in the subsequent signal pre-processing.

### 2.3. Experimental paradigms

**FIGURE 1:**
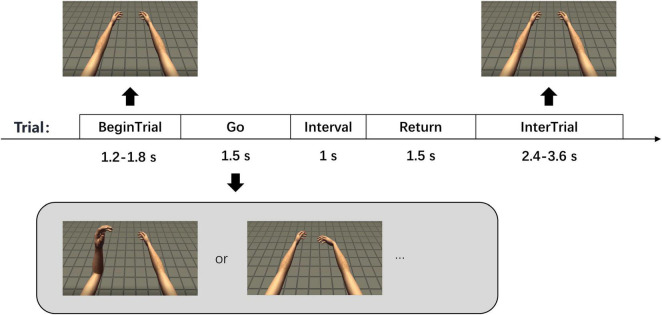
Experimental paradigms. There are 5 phases in each trial with corresponding time shown in the figure. The two pictures at the bottom show two of the seven tasks in the experiment, which are left elbow flexion and right wrist flexion.

In the experiment, a screen was placed in front of the participant. A model (FPS Handy Hands, Unity Asset Store) of the left and right upper limbs was presented on the screen, including hands, forearms and upper arms. The model was created based on the real morphology of arms and positioned from a first-person perspective. The participant could imagine this model as his own upper limbs, which was conducive to motor imagery (Figure 1).

There were 7 motor imagery tasks in the experiment, which were rest, left elbow flexion, right elbow flexion, bilateral elbow flexion, left wrist flexion, right wrist flexion, and bilateral wrist flexion. 3-category, 4-category, and 7-category experiments were carried out throughout the whole experiment period and only 4-category and 7-category data were used in this study. The 3-category experiment included 3 kinds of tasks of rest, left and right wrist flexion. The 4-category experiment included 4 kinds of tasks of rest, left, right and bilateral elbow flexion. The 7-category experiment included all tasks. The number of each task was the same in a session and all tasks appeared randomly. A total of 1–4 sessions were carried out per day according to the status of the participant. A total of 32 sessions with 2,215 trials of signals were collected.

Each trial had 5 phases in the experiment, which were “Begin Trial,” “Go,” “Interval,” “Return,” and “Inter Trial” (Figure 1). The last 800 ms in “Begin Trial” phase was called “Prepare” phase. In the “Begin Trial” phase, the arm model was placed naturally. In the “Go” phase, the model moved slowly with constant velocity until it reached the maximum angle of flexion according to the current type of movement. We mainly explored the neural signals of motor imagery, regardless of the velocity. The use of constant velocity not only eliminates the effect of velocity change, but also facilitates the motion setting of the model. In the “Interval” phase, the model remained at the maximum flexion angle, and then slowly returned to the initial position in the “Return” phase. In the “Inter Trial” phase, the model stayed in the initial position again and waited for the next trial. There was also text above the arm model to indicate the movement phase and task. The participant remained relaxed in the “Begin Trial” and “Inter Trial” phases and performed the corresponding motor imagery while watching the movement of the arm model in the other three phases. In the rest task, the arm model remained stationary in all 5 phases and the participant remained relaxed. The durations of “Begin Trial” and “Inter Trial” phases were not fixed, which were 1.2–1.8 s and 2.4–3.6 s, respectively. The durations of “Go,” “Interval” and “Return” phases were 1.5, 1, and 1.5 s, respectively.

### 2.4. Signal pre-processing

The raw data recorded in the experiment were first filtered by an eight order Butterworth bandpass filter between 0.3 and 480 Hz offline to obtain valid LFP signals. The data from 800 ms before (“Prepare” phase) to 1,500 ms after (“Go” phase) the movement onset were used. Then signals with obvious artifacts in all trials and channels were selected through visual observation. The artifacts may be caused by sudden interference or poor quality electrodes, which were shown in Figure 7. In the subsequent neural decoding, the input features of these contaminated signals were set to 0, and in other analysis, these signals were directly excluded.

Data was analyzed using Matlab and Python, the dPCA code referred to Kobak’s code (Kobak et al., 2016).

### 2.5. Analysis of spectrogram

**FIGURE 2:**
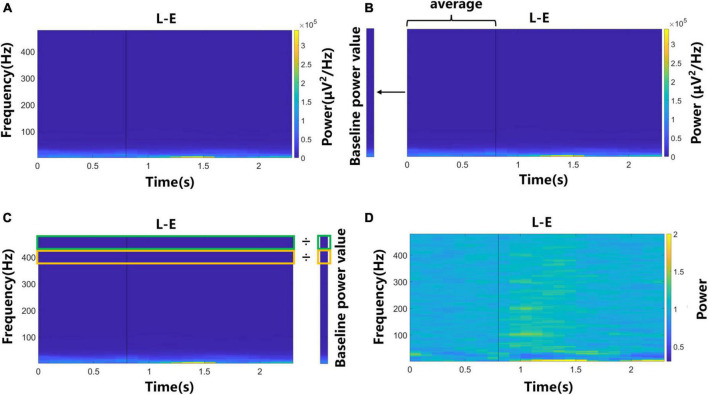
The flow chart of the normalization process of spectrograms. Data of left elbow motor imagery trials were used. **(A)** The averaged spectrogram across all trials and channels. **(B)** Baseline power value was calculated by averaging the power of each frequency value in the “Prepare” phase. **(C)** Each power value in the spectrogram was divided by the baseline power value of the corresponding frequency. **(D)** The normalized spectrogram.

The LFP signal of each trial was divided into several 300 ms windows with 100 ms overlap. The multitaper power spectral density estimate method was used for the signal of each window to obtain spectrogram. To observe the power change of signals during motor imagery more clearly, the spectrograms were normalized. Figure 2 shows each step and its output. For each task, first, the averaged spectrogram across all trials and channels was obtained (Figure 2A). Then we calculated the average power of each frequency value in the “Prepare” phase, as the baseline power value of this frequency value (Figure 2B). Next, each power value in the spectrogram was divided by the baseline power value of the corresponding frequency to obtain the normalized spectrogram (Figures 2C, D). In this study, the average power change over time of a certain frequency band in the normalized spectrogram was called the average power signal, which had 23 values in a trial and would be used in the following analysis.

### 2.6. Data structure for DPCA

A common dimensionality reduction method for multi-label high-dimensional data is dPCA. It maximizes the variance of the original high-dimensional neural data for each parameter according to different parameter types (Kobak et al., 2016). The average power signal was used for the analysis of dPCA. Before applying dPCA, a data set had to be created, which including all the information of channels, laterality, regions, time and repetition times. The structure of the data set was Data = C*L*R*T*M, where C represented the number of channels (*C* = 96), L represented laterality (*L* = 3), which were ipsilateral, contralateral and bilateral sides, R represented regions (*R* = 2), which were elbow and wrist, T represented the number of time windows of the average power signal (*T* = 23) and M represented the number of repetitions of each task in a session, which was determined according to the experimental paradigm.

### 2.7. Feature extraction and classification

The average power signals of “Go” phases were used as the features for decoding. We gathered all the features of 96 channels together as the features of a trial. Leave-one-out cross validation method, which could divide most of the data into the training set and was suitable for classification tasks with small sample sizes, was used for the neural signal decoding. Suppose there are n samples, leave-one-out cross validation takes turns using each sample as the testing sample and the other n-1 samples as training samples. In the calculation process, n classifiers and n classification results can be obtained, and then these n results are averaged to get the final classification accuracy. The decoding result calculated by this method is deterministic due to the fixed division of the training set and testing set.

The classification algorithm used in this study is random forest, which has the characteristics of high accuracy, simple implementation process and strong anti-overfitting ability. Furthermore, the capacity of random forest to deal with data with many features is suitable for the data set in this study.

## 3. Results

### 3.1. Power change in spectrograms

**FIGURE 3:**
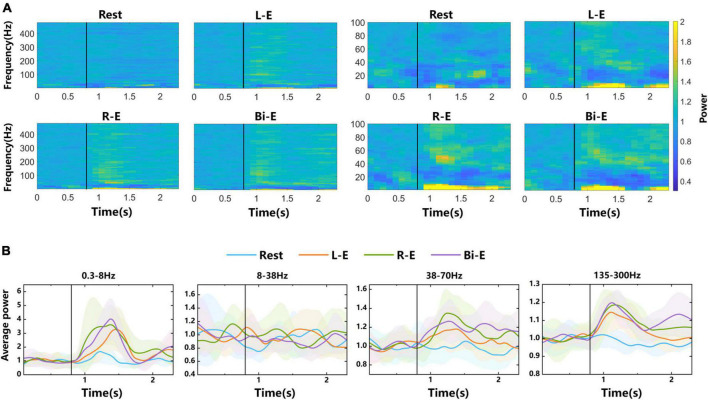
Time-frequency analysis of LFP signals from 4-category experiments. **(A)** Spectrograms of LFP signals. The labels “Rest”, “L-E”, “R-E”, and “Bi-E” in the graphs represent rest task and left, right and bilateral elbow flexion motor imagery tasks, respectively. The left four small graphs show the overall power changes of the 0.3–480 Hz frequency band, whereas the right four small graphs highlight the details of power changes in the 0.3–100 Hz frequency band. Each graph is an average normalized spectrogram of all trials of the corresponding task on all electrodes. **(B)** The average power curves over time in 0.3–8, 8–38, 38–70, and 135–300 Hz frequency bands. The shaded areas are error bands. The vertical black lines represent the time of the movement onset.

Spectrograms were drawn to observe the power changes of different frequency components of LFP signals before and during rest, left, right, and bilateral elbow flexion tasks in Figure 3A. Each small graph shows an average spectrogram across all channels and trials, and the power values are normalized (see section “2. Materials and methods”). The vertical black lines represent the time of movement onset. The power change of rest task was relatively unobvious in the spectrogram, whereas in the condition of three motion tasks, the power changed obviously after movement onsets. The modulation for the rest might be caused by sensory input. In the “Go” phase, there was text prompt on the screen. In the beginning of the “Go” phase, there was sound prompt. They all prompted the subsequent movement tasks. The frequency range of <8 Hz and >38 Hz showed power enhancement, whereas the range of 8–38 Hz showed power suppression. Combined with the division of frequency bands in other studies (Jin et al., 2016), the LFP signal was partitioned into 5 frequency bands, which were 0.3–8, 8–38, 38–70, 70–135, and 135–300 Hz, for the following analysis.

The average power changes over time in 0.3–8, 8–38, 38–70, and 135–300 Hz frequency bands were plotted in Figure 3B. Among them, the curves of 0.3–8, 38–70, and 135–300 Hz frequency bands have obvious peaks after movement onsets in motion tasks. In the 8–38 Hz frequency band, average power is suppressed. The error bands of average power curves in different tasks overlap a lot with unobvious distinction. Since it was explored in the literature that the high frequency band of LFP signal contained more information (Li et al., 2014), this study mainly focused on the bands of high frequency and with obvious power changes.

### 3.2. Energy in different movements

**FIGURE 4:**
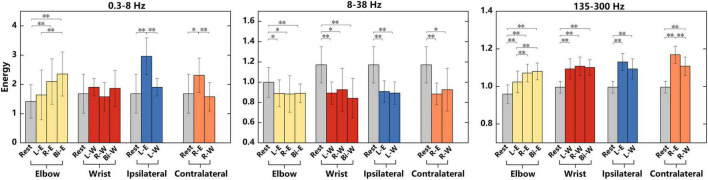
Significant differences in the average energy of “Go” phase under different tasks in 0.3–8, 8–38, and 135–300 Hz frequency bands. “L-W”, “R-W”, and “Bi-W” represent left, right and bilateral wrist motor imagery, respectively. All tasks were divided into four groups of elbow, wrist, ipsilateral and contralateral motor imagery. Significant differences between the tasks in each group were analyzed. The data used for the analysis of elbow group were from the 4-category experiments, and data used for the other three groups were from the 7-category experiments. Significant differences analysis was performed by ANOVA, *represents *p* < 0.05, ^**^represents *p* < 0.01.

To conduct a more specific analysis of the energy difference under different tasks, the average energy of “Go” phase in different frequency bands were calculated in Figure 4. The recorded data were divided into four groups, namely, elbow, wrist, ipsilateral and contralateral motor imagery. The analysis of variance (ANOVA) was used to analyze the significant differences of average energy under different tasks in each group. The elbow and wrist groups explored the energy difference between different lateral tasks, whereas the ipsilateral and contralateral groups explored the difference between different regions. In Figure 4, the energy of 0.3–8, 8–38, and 135–300 Hz frequency bands were analyzed respectively. Among the three frequency bands, the energy of signals in the 135–300 Hz band had the best discrimination for different tasks. What’s more, the elbow motions of different laterality were easier to distinguish than wrist motions. When performing motor imagery, the energy in 8–38 Hz band reduced obviously compared with rest state, however, no significant differences were shown between two motion tasks. The information related to kinematics might be suppressed in this band. The *p*-values of ANOVA were shown in Table 1.

**TABLE 1:** The *p*-values of the significant differences analysis in Figure 4.

Frequency band/Hz	Group	*P*-value					
0.3–8	Elbow	0.3344	0.0030	8.5806 × 10^–5^	0.0838	0.0086	0.2966
	Wrist	0.3729	0.6857	0.5326	0.0982	0.8676	0.2585
	Ipsilateral	3.3650 × 10^–4^	0.3729	2.6423 × 10^–4^			
	Contralateral	0.0381	0.6857	0.0069			
8–38	Elbow	0.0193	0.0331	0.0097	0.8930	0.9901	0.8764
	Wrist	9.0419 × 10^–4^	0.0118	0.0010	0.6837	0.4975	0.3683
	Ipsilateral	8.5017 × 10^–4^	9.0419 × 10^–4^	0.7570			
	Contralateral	3.8641 × 10^–4^	0.0118	0.5799			
135–300	Elbow	4.8727 × 10^–4^	7.4396 × 10^–9^	1.1784 × 10^–9^	0.0070	0.0017	0.5547
	Wrist	1.4147 × 10^–4^	9.7873 × 10^–6^	5.1440 × 10^–6^	0.5503	0.7300	0.7454
	Ipsilateral	4.2757 × 10^–4^	1.4147 × 10^–4^	0.1227			
	Contralateral	1.1608 × 10^–4^	9.7873 × 10^–6^	0.0099			

The significant differences were analyzed by ANOVA. The six *p*-values from left to right in elbow group represent the differences of rest and left elbow task, rest and right elbow task, rest and bilateral elbow task, left elbow and right elbow task, left elbow and bilateral elbow task, right elbow and bilateral elbow task. The order of *p*-values in other groups is similar.

We also performed the analysis in Figure 4 on the signals of “Prepare” phase and found that there were only two conditions that had significant differences. One was the significant difference between rest and left elbow flexion task in the analysis of elbow group using the energy of 8–38 Hz frequency band (ANOVA, *p* < 0.05); the other was between rest and right wrist flexion task in the analysis of ipsilateral group using the energy of 135–300 Hz frequency band (ANOVA, *p* < 0.05). Therefore, a lot of energy changes only occurred during the “Go” phase.

Now that we proved that LFP signals of unilateral cerebral cortex had different representations during motor imagery not only on different sides, but also on different body regions, especially in the 135–300 Hz frequency band. To explore whether the representations of body regions and laterality were in different dimensions, we applied dPCA to reduce the dimensionality of data (see section “2. Materials and methods”).

**FIGURE 5:**
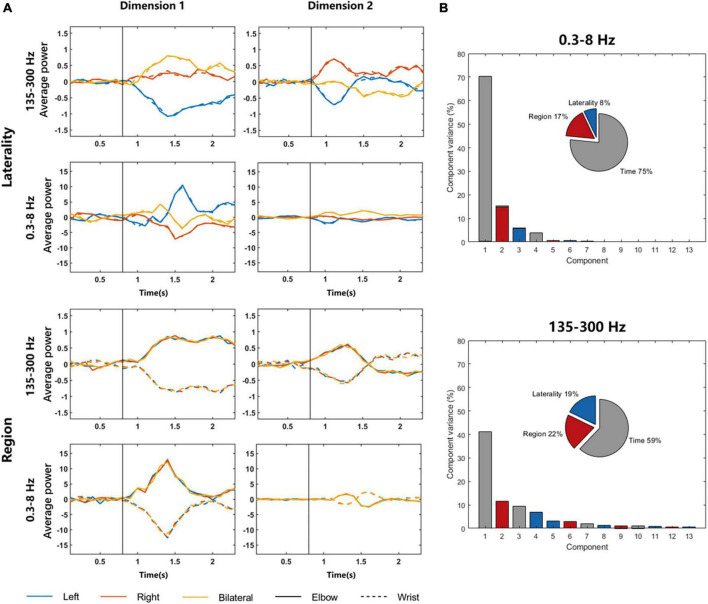
DPCA results of LFP signals in 0.3–8 and 135–300 Hz frequency bands. **(A)** The graphs in the left column are components with the largest variance, and the graphs in the right column are components with the second largest variance. The four graphs above show the dimension related to laterality, and the four graphs below show the dimension related to regions. The solid and dotted lines in the graphs represent LFP signals during elbow and wrist motor imagery, respectively. The vertical black lines represent the time of the movement onset. **(B)** The variance of three dimensions.

Figure 5A shows the dimensionality reduction results for different parameters. The graphs in the left column are components with the largest variance, and the graphs in the right column are components with the second largest variance. The four graphs above show the dimension related to laterality, and it can be found that the signal difference between elbow and wrist is small, but the signals of different sides can be clearly distinguished. *T-*test was used to explore the significant differences of 135–300 Hz frequency band in dimension 1. The *p*-values representing the differences of left elbow and right elbow task, left elbow and bilateral elbow task, right elbow and bilateral elbow task were 6.8437 × 10^–11^, 2.7506 × 10^–11^ and 0.0011, respectively. The *p*-value representing the difference of left elbow and left wrist task was 0.9908. The four graphs below show the dimension related to regions. The elbow and wrist signals can be clearly distinguished, whereas the left, right, and bilateral signals are all mixed together. The *p*-value representing the difference of left elbow and left wrist task of 135–300 Hz frequency band in dimension 1 was 6.3854 × 10^–13^. In addition, the dPCA results of 135–300 Hz frequency band signals are more distinguishable than that of 0.3–8 Hz signals. For example, compared to the *p*-values listed above, the *p*-values of 0.3–8 Hz frequency band in dimension 1 representing the differences of left elbow and right elbow task, left elbow and bilateral elbow task, right elbow and bilateral elbow task were 1.4105 × 10^–14^, 0.0557 and 0.0012, respectively. The results demonstrated that there existed region-related dimension for distinguishing the moving region, and the laterality-related dimension to identify which side of the limb was in motion. Figure 5B shows the percentage of overall variance which the three dimensions capture. The different conditions significantly separated in these dimensions and the variance of region dimension was larger than laterality dimension.

### 3.3. Decoding performance

**FIGURE 6:**
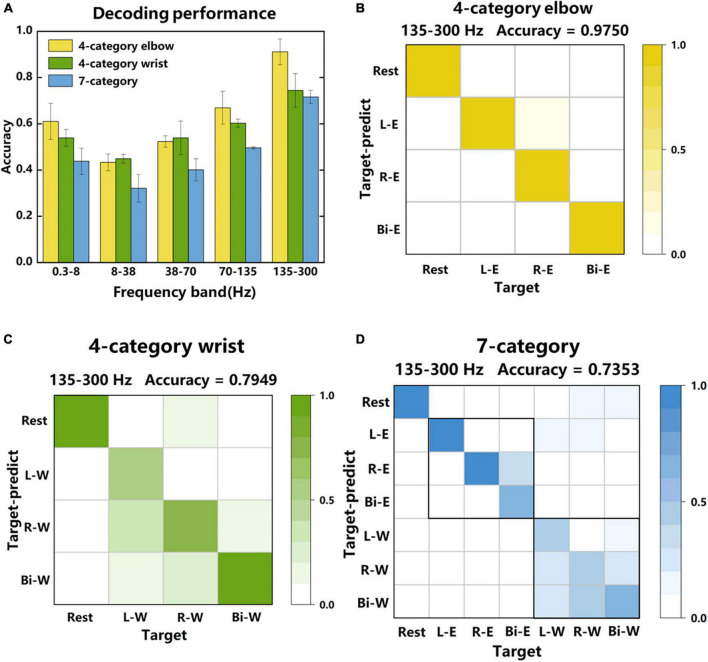
Decoding performance of LFP signals in different frequency bands. **(A)** The classification accuracy of 4-category elbow, 4-category wrist, and 7-category tasks in different frequency bands. The 4-category elbow tasks are rest, left, right and bilateral elbow flexion motor imagery. The 4-category wrist tasks are rest, left, right and bilateral wrist flexion motor imagery. The 7-category tasks contain all tasks. **(B)** The specific classification results of each category of one session in the 135–300 Hz frequency band of the 4-category elbow tasks. The data were obtained from 4-category experiments. **(C,D)** The specific classification results of 4-category wrist and 7-category tasks. The data were obtained from 7-category experiments. The accuracy indicated on the upper right are average accuracy of each category.

In Figure 6, average power signals (see section “2. Materials and methods”) in the “Go” phase were used as features to classify different tasks, and the classification accuracy reflected the richness of the motion information in the signal to some extent. Figure 6A shows the decoding performance of 4-category elbow (left, right and bilateral elbow flexion), 4-category wrist (left, right and bilateral wrist flexion) and 7-category tasks. For comparison, signals from 5 frequency bands were extracted for classification. The highest accuracy reached to 0.91 ± 0.06, 0.74 ± 0.07, and 0.72 ± 0.03 in the 135–300 Hz frequency band, respectively. These results showed that the LFP signal not only performed well in the decoding of different sides, but also had a good performance in mixed tasks with different laterality and regions. In the low frequency band of 0.3–8 Hz, there were also relatively good decoding performances, but in the power suppression band of 8–38 Hz, the accuracies were the lowest.

**FIGURE 7:**
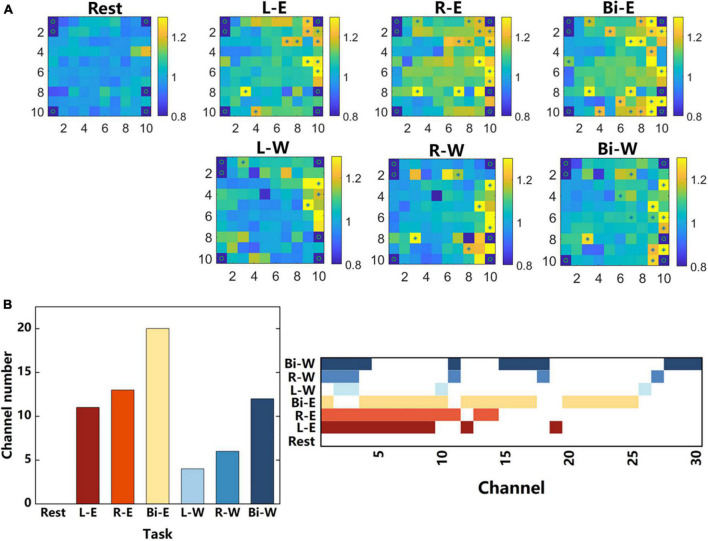
Single-channel analysis. **(A)** All the channels were arranged according to the actual distribution on the electrode array, and the average energy of the “Go” phase and “Prepare” phase of each channel were calculated, averaged across all trials during each task. The color in every small square represents the ratio of the average energy of the “Go” phase to that of the “Prepare” phase. “*” Represents significant difference in the energy of the two phases on the corresponding electrode (ANOVA, *p* < 0.01), and “o” represents a channel that does not exist or has poor signal quality. The signal data came from the 135–300 Hz frequency band of the 7-category tasks. **(B)** The number of channels with significant differences in different tasks and the activation state of all these channels under different tasks.

Figures 6B–D further shows the specific classification results of one session in the three classification tasks, respectively. The 135–300 Hz frequency band signals were used since they led to the highest accuracies. For 4-category elbow task, 301 trees were used in the random forest classifier, and the average layer and node of trees were 4 and 12. For 4-category wrist task, 95 trees were used, and the average layer and node of trees were 4 and 14. For 7-category task, 262 trees were used, and the average layer and node of trees were 7 and 30. The accuracy of 4-category elbow (B) was higher than that of 4-category wrist (C), and for the 7-category task (D), the errors in elbow tasks were fewer than that in wrist tasks. Therefore, elbow tasks were easier to distinguish than wrist tasks in this study. What’s more, contralateral and bilateral tasks were most likely to be confused and brought the decoding performance down.

In general, the results demonstrated the feasibility of using LFP signals of unilateral cerebral cortex to decode motor imagery tasks of different laterality and regions, and contralateral and bilateral movements were the easiest to confuse.

### 3.4. Single-channel analysis

For signals recorded by multi-channel electrode arrays, analyzing single-channel signals could often get more detailed information than averaging the signals of all channels. We also compared the classification accuracies using the features obtained by two methods in this study. One of the methods was averaging the average power signals obtained from the 96 channels on the array as the features, and the other one was splicing the average power signals of all channels, which was the method used in the previous decoding analysis. In all frequency bands, the accuracy of the latter methods were mostly higher than that of the former methods. Therefore, the signal recorded by each channel in the electrode array was further analyzed.

Figure 7A shows the ratios of the average energy in 135–300 Hz frequency band of “Go” phase to that of “Prepare” phase for each channel under different tasks and identifies the channels with significant differences (ANOVA) in energy in the two phases. In general, during motor imagery, only a small number of channels had significant energy activation compared with the rest state, and they were concentrated on the right side of the array in this study. Comparing the energy activation while performing motor imagery in different body regions, it could be found that the energy activation of the elbow was larger than that of the wrist, and the number of channels with significant differences was more than that of the wrist. During the motor imagery of elbows, there was obvious activation in the upper right corner of the array, but there was no such activation during the motor imagery of wrists. The location of the upper right corner is closer to the midline and closer to the central sulcus. Comparing the energy activation between different lateral motor imagery, it could be found that the motor imagery of the right and bilateral sides had obvious activation in the lower right corner, but there was no such activation during the motor imagery of the left side.

Figure 7B shows the number of channels with significant differences and the activation state of all these channels under different tasks. It could be found that the activation channels of the same region had a great overlap when two unilateral movements were performed, and the range of significant activation during bilateral movement was larger than the superposition of two unilateral movements.

While performing motor imagery, there were only 30 channels that had significant differences in energy compared to the rest state. Did these channels contain most of the motion information? We further tested whether the classification accuracy obtained using only the features of these 30 channels was equivalent to the original one. As shown in the section “2. Materials and methods”, we also used the average power signals of “Go” phases (15 values) of a specific frequency band as the features extracted from one channel. We gathered the features of 30 channels as the input of the classifier. The 135–300 Hz signal with the highest classification accuracy in the 7-category tasks was used, and the classification accuracy obtained by using only the features of these 30 motion-related channels was 0.824. Compared with the accuracy rate, 0.735, obtained by classifying the features of all channels, only considering the features of motion-related channels could get better decoding performance. It could be seen that during motor imagery, several channels contained a large amount of motion information and could be used to achieve better decoding results. This was of great significance for optimizing the brain computer interface algorithm and improving the decoding performance.

**FIGURE 8:**
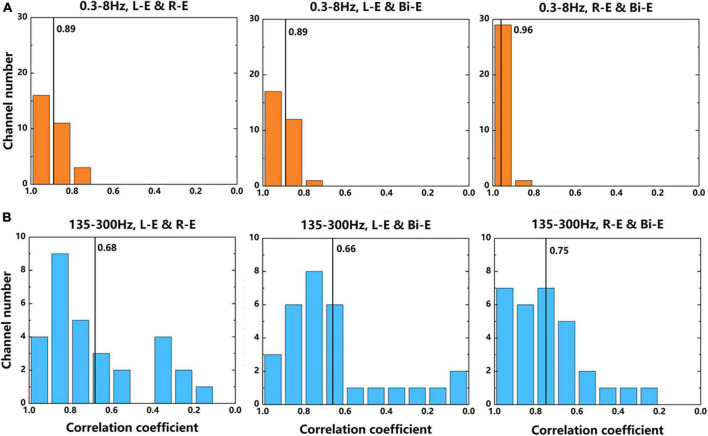
The number of channels corresponding to each correlation coefficient between two movements. **(A)** The results of signals in 0.3–8 Hz frequency band. **(B)** The results of signals in 135–300 Hz frequency band. The average power signals from the 7-category experiment were used to calculate the correlation coefficients. The numbers near the black lines in the graphs represent the average correlation coefficients of all 30 channels.

The 30 channels that had significant activation while moving compared with rest state were further analyzed by comparing the correlation of signals between different lateral movements of elbows. The data used were average power signals of “Prepare” and “Go” phases in the frequency bands of 0.3–8 and 135–300 Hz of 7-category experiment. The absolute correlation coefficient of each channel between two movements was calculated. Figure 8 shows the number of channels corresponding to each correlation coefficient. It could be seen that in the two frequency bands, the correlation coefficients of the contralateral and bilateral movements were the largest. This further illustrated that the activation patterns of contralateral and bilateral arms during motor imagery were the most similar. For the same two tasks, the correlation coefficients of the 0.3–8 Hz frequency band were larger than that of the 135–300 Hz band. It showed that the low frequency signals were more similar, which was consistent with the results that the accuracy of the decoder using low frequency signal was lower than that of the high frequency signal.

## 4. Discussion

In this study, LFP signals recorded from unilateral cerebral cortex were analyzed for the representation and decoding of arm motor imagery in different sides and regions. The results showed that in 135–300 Hz frequency band, there were significant differences in the power of LFP signals between tasks of different laterality and regions, and the lateral and regional information were characterized in two dimensions. Among all frequency bands, the 135–300 Hz band signal had the best decoding performance. The movements could be accurately distinguished even in the 7-category tasks mixing with different laterality and regions, and the confusion mainly occurred between contralateral and bilateral movements. The low frequency band signal of 0.3–8 Hz also had relatively high accuracy. From the analysis of the single-channel LFP signals, it could be seen that the activation intensity and range of different movements were not the same, whereas the LFP signals of contralateral and bilateral arm movements were more similar. What’s more, using the features of motion-related channels only could improve the decoding performance.

### 4.1. The representation of different lateral movements using unilateral LFP signals

The origin of ipsilateral signals in the cerebral cortex has its structural basis. Current research (Nyberg-Hansen and Rinvik, 1963; Brus-Ramer et al., 2009) generally believes that the activation of the ipsilateral cortex is related to the corticospinal tract and the corpus callosum. Although most of the neural fibers in the corticospinal tract project to the contralateral cortex, a small part of them still projects directly to the ipsilateral cortex (Lacroix et al., 2004). The corpus callosum connects the corresponding parts of the cerebral hemispheres on both sides, so that the activation of the contralateral cortex can affect the ipsilateral cortex (Brus-Ramer et al., 2009). Therefore, the kinematic information of both the ipsilateral and contralateral movements can be reflected in the ipsilateral cortex. In the bilateral movement, the signals from both sides may affect each other. This lays the foundation for the representation and decoding of the contralateral, ipsilateral and bilateral movements based on the ipsilateral neural signals. What’s more, the bilateral representation ability might also related to the dominance of left hemisphere since the electrode array was implanted in the left M1.

According to the activation in the electrode array and the number of channels that had significantly different energy between the motor imagery and rest state in Figure 7, although the representation of the contralateral and ipsilateral movements in the motor cortex had some similarity, there were also obvious differences between them. Many channels activated both during the contralateral and ipsilateral movements, but a few channels activated only during one of the movements. The neurons involved in the two movements might not be exactly the same. Previous studies have also reported that although the contralateral and ipsilateral signals could decode the movement of the same limb, they were still very different at the level of single-neuron signals (Ames and Churchland, 2019). In this study, we showed the differences in neural signals between the contralateral, ipsilateral and bilateral motor imagery, at the level of single-channel LFP signals.

Donchin et al. (1998) used single unit activity to find that in the M1 area, more neurons were directionally selected during contralateral hand movement than ipsilateral hand movement, and more neurons exhibited activity in bilateral movement than unilateral movement. There was also the similar condition in the number of significantly activated channels of the LFP signal of different lateral movements (Figure 7B). This also indicated that bilateral movement was not a superimposition of two unilateral movements. Donchin et al. (2002) got the similar conclusion using spikes by comparing the neural signals of bilateral movement and the similar movement of one arm while the other was stationary. Some LFP signal channels were only related to bilateral movement and might have some coordination functions in the bilateral movement. Realizing the specific role of these neurons played needed further research. Moreover, according to the correlation of neural signals during different lateral movements, it could be found that the neural signals of bilateral and contralateral movements were more similar. This might because that the cerebral cortex mainly controlled the contralateral limb, which was in line with the classic theory of the cross control from the brain to limbs.

It could be found In Figure 5 that the LFP signals represented the laterality and regions of movement in two dimensions. There were similar results in the study of Willett et al. (2020), which were obtained using spike signals. Willett et al. (2020) found that the movement and laterality were distributed in two independent dimensions, and thus proposed a compositional coding hypothesis indicated as movement-coding and effector-coding dimensions. Our research proved this hypothesis from the perspective of LFP signals and at the same time supplemented it. Willett et al. (2020) only explored that in the lateral dimension, the spike signals of ipsilateral and contralateral movements could be well distinguished. Our research found that even if bilateral signals were added, these three types of lateral signals were distinguishable. This demonstrated that in the coding of movement laterality, not only single limb could be coded, the simultaneous movement of multiple limbs could also be distinguished. In general, based on the compositional coding hypothesis, the representations of different lateral movements had a certain similarity because of the code of movement regions, whereas the code of movement laterality made them possible to distinguish.

However, since the participant was a long-term paralyzed patient and had no voluntary arm movement, it was inevitable that there existed neural plasticity changes in his brain, which could affect the conclusion of the experiment. More participants may be recruited to rule out this possibility in the future.

### 4.2. Decoding analysis of different frequency bands

In this study, spectrograms of the LFP signals were drawn and decoding performance in different frequency bands were calculated. In the spectrograms, different power characteristics of enhancement or suppression were shown in different frequency bands. The pattern was similar to that obtained by Rickert et al. (2005) using the LFP signals obtained during the center-out tasks of monkeys. In terms of decoding performance in different frequency bands, many studies have found that the decoding ability of alpha and beta frequency bands was poor, whereas low frequency (<5 Hz) and high frequency (>50 Hz) bands could decode a lot of kinematic information (Jackson and Hall, 2017). This was consistent with the decoding performance of different frequency bands in this study, and our study supplemented the comparison in LFP signals from human during motor imagery.

Rickert et al. (2005) also found that the LFP activity was not modulated with the direction of movement in the ≈30 Hz frequency band with reduced amplitude in the spectrogram, but obvious cosine-like tuning could be seen in other frequency bands with increased amplitude. Miller et al. (2010) also found in the ECoG signals from human that in the 8–32 Hz frequency band, the ranges of energy drop caused by the motor imagery of the hand and tongue were wide and overlapping, in contrast, the energy increase ranges of higher frequency band were concentrated and non-overlapping. Therefore, we considered that the information related to kinematics might be suppressed in the 8–38 Hz frequency band. The signal contained some general motion information, rather than specific motion details such as laterality and regions. This also explained why alpha and beta frequency bands were commonly used for EEG signals when studying motor imagery, but did not perform well in LFP decoding. In the decoding of EEG signals, whether a body part moved was often took into consideration, and no specific kinematic information or the distinction of moving parts in a small area was involved.

In this study, the average power signal of the high frequency band (135–300 Hz) had the best discrimination for different laterality and regions of the motor imagery (Figure 4) and had the best classification accuracy in the 4-category and 7-category tasks (Figure 6). In previous study, high frequency band LFP signals also had good performance in the decoding of unilateral movement of monkeys. Zhuang et al. (2010) divided the frequency range of 0.3–400 Hz into 7 different bands and studied the decoding performance of these bands on the kinematic information of monkeys when they freely reached and grasped moving objects. The results showed that the neural decoding of the broad high-frequency LFP band (200–400 Hz) exceeded all other bands. Therefore, the high-frequency LFP signal might contain more kinematics information. In addition, in the 7-category task, the decoding performance of the wrist was not as good as the elbow. This might be due to that the electrode array covered more arm area in the motor cortex instead of wrist or hand area. This was also reflected in the activation diagram of the single-channel electrode (Figure 7A). The activation of the elbow was greater than that of the wrist during motor imagery.

Low-frequency (0.3–8 Hz) signals also had relatively good decoding performance in this study. Liu et al. (2020) found that the movement-related cortical potential (MRCP) signals at C3 and C4, filtered by 0.01–3 Hz bandpass filter, both showed different characteristics when the left hand and right hand moved, which indicated that the MRCP of the unilateral cerebral cortex carried some lateral information. However, few studies have distinguished between the signals during bilateral and unilateral movements. In addition, Gunduz et al. (2016) studied ECoG signals during 8-target center-out movement task and found that the classification accuracy based on high-frequency signals was higher than that of low-frequency signals, and did not improve by adding low-frequency signal features. Therefore, the low-frequency signals of unilateral brain cortex might be able to distinguish the ipsilateral and contralateral movements, but the detailed information of the movement in them were not so much as the high-frequency signals.

Since the signals of different frequency bands have different physiological processes behind them, and they also represent different information, choosing a suitable frequency band of LFP signals can effectively improve the performance of BCI. For further interpretation of signals in various frequency bands, more research in neuroscience and other fields is needed.

### 4.3. Bilateral BCI based on unilateral LFP signal

Our results proved the feasibility of bilateral BCI based on the unilateral LFP signal, which broadened the application of BCI technology. First, it is possible to control bilateral prostheses or machines to move based on unilateral signals. In this way, the convenience of the BCI system for patients with bilateral limb disorders can be further increased without increasing the injury. Second, the LFP signal itself has many advantages. Compared with spikes, it has low bandwidth and can maintain high stability and decoding accuracy for a long period of time. These features make it possible for small, low power consumption and long-term BCI applications.

However, the bilateral movement in this study was somewhat simple and the two arms were studied as two separate parts. The coordination of the arms, which plays an important role in daily life, has not been studied in depth. Downey et al. (2020) explored the ability for human to control two virtual arms. The neural mechanism of the coordinated movement of bilateral limbs is not clear enough, and the neural signal processing method that can realize the coordinated movement of the external device need further study. Furthermore, to make BCI restore a more naturalistic motor performance, it is relevant to include a key cognitive component of action control, for example, inhibitory control (Mirabella, 2014). Such a need had been suggested previously in the framework of the evolution of newer BCIs capable of decoding the neural activity underpinning decision-making processes bringing to goal-directed actions (Mirabella and Lebedev, 2017). Interestingly, it has been recently shown that even one domain of motor inhibitory control (Bari and Robbins, 2013), for example, reactive inhibition, the ability to stop outright a movement at the presentation of a stop signal, might be coded mainly by the left hemisphere (Mancini and Mirabella, 2021). Thus, recording neural activity from the M1 of the left hemisphere, as was done in the present study, might potentially represent an optimal solution for providing more effective BCI. How to implement a BCI system that can smoothly control bilateral prostheses or devices and put it into use is still an important topic, and a lot of efforts from various fields are needed.

## Data availability statement

The raw data supporting the conclusions of this article will be made available by the authors, without undue reservation.

## Ethics statement

The studies involving human participants were reviewed and approved by the Institutional Review Board for Human Studies of the Second Affiliated Hospital of Zhejiang University School of Medicine. The patients/participants provided their written informed consent to participate in this study.

## Author contributions

JL, DL, and ZW contributed to the experiment conduct and data processing. JL wrote the manuscript. JZhu and JZha designed and completed the electrode implantation surgery. LF, YW, and KX contributed to the experimental system design and the conception of the study. All authors contributed to manuscript revision, read, and approved the submitted version.
